# CD47 is a negative regulator of intestinal epithelial cell self-renewal following DSS-induced experimental colitis

**DOI:** 10.1038/s41598-020-67152-w

**Published:** 2020-06-23

**Authors:** Yueqin He, Xinlei Sun, Weiwei Rong, Rong Yang, Hongwei Liang, Ying Qi, Limin Li, Ke Zen

**Affiliations:** 1 Nanjing University Advanced Institute of Life Sciences, Nanjing, China; 2 Jiangsu Engineering Research Center for microRNA Biology and Biotechnology, Nanjing University, Nanjing, Jiangsu 210093 China; 3 Department of Gastroenterology and Hepatology, Jinling Hospital, Medical School of Nanjing University, Nanjing, China

**Keywords:** Growth factor signalling, Immunological disorders

## Abstract

CD47 deficient mice are resistant to dextran sulfate sodium (DSS)-induced experimental colitis. The underlying mechanism, however, remains incompletely understood. In this study, we characterized the role of CD47 in modulating homeostasis of gastrointestinal tract. We found that CD47 expression in both human and mouse intestinal epithelium was upregulated in colitic condition compared to that under normal condition. In line with this, CD47 deficiency protected mice from DSS-induced colitis. Analysis based on both intestinal organoid and cultured cell assays showed that CD47 deficiency accelerated intestinal epithelial cell proliferation and migration. Mechanistically, western blot and functional assays indicated that CD47 deficiency promoting mouse intestinal epithelial cell proliferation and migration follow cell injury is likely through upregulating expression of four Yamanaka transcriptional factors Oct4, Sox2, Klf4 and c-Myc (OSKM in abbreviation). Our studies thus reveal CD47 as a negative regulator in intestinal epithelial cell renewal during colitis through downregulating OSKM transcriptional factors.

## Introduction

Gastrointestinal epithelium is a unique system maintained by the balance between apoptosis and renewal of intestinal epithelial cells (IECs). In general, the renewal of the entire intestinal epithelium occurs every 3 to 5 days^1^. Inflammatory bowel disease (IBD), including ulcerative colitis (UC) and Crohn’s disease (CD), is characterized pathologically by intestinal inflammation and epithelial injury^2,3^. Increased intestinal epithelial cell (IEC) apoptosis has been detected in acute inflammatory colonic tissue from the biopsies of patients with UC and CD^4^. In the past, much attention has focused on how to suppress the inflammation-associated cell apoptosis of IECs to maintain the gastrointestinal epithelium homeostasis^5,6^. It has been reported that neutralization of inflammatory cytokines including TNFα and IFNγ can reduce epithelial apoptosis^7,8^. Opposite to epithelial cell apoptosis, cell self-renewal also plays a key role in the recovery of IBD. However, the mechanism of regulating intestinal epithelial cells proliferation and wound repair in inflammatory bowel disease is still incompletely understood.

CD47 is a widely expressed transmembrane protein in various cells^9^. Besides serving as a self-recognition molecule that prevents cell phagocytosis by macrophages and other phagocytes via binding to its counter-receptor SIRPα^10^, CD47 itself also acts as a signaling molecule in modulating various cell functional aspects^11^. For example, CD47 has been identified as a repressor of stem cell self-renewal in normal cells by limiting c-Myc level, and that knockdown of CD47 enhanced the proliferation of endothelial progenitor cell, mouse lung endothelial cells and renal tubular epithelial cells^12–14^. Previous studies also reported CD47-deficient (CD47^−/−^) mice were resistant to 2% dextran sulfate sodium (DSS)-induced experimental colitis^15^ and TNBS-induced colitis^16^. Although chronic inflammation with less neutrophil production or lower percentage of SIRPα^+^CD103^−^ DCs and Th17 responses have been suggested to contribute to the resistance of CD47^−/−^ mice to experimental colitis, the underlying mechanism remains elusive. It is not clear whether CD47 play a role in modulating epithelial cell renewal following the DSS-induced epithelial injury.

In the present study, we found that CD47 expression is significantly increased in patients with UC and CD as well as in mouse experimental colitis models. The CD47 expression in IECs is correlated with the severity of colitis, and CD47 deficiency protects mice from DSS-induced colitis. Our studies have further shown that CD47 inhibits IEC proliferation and migration likely through suppressing four Yamanaka transcriptional factors (abbreviated as OSKM), octamer-binding transcription factor 4 (Oct4), sex-determining region Y-box 2 (Sox2), Krüppel-like factor 4 (Klf4), and the cellular homolog of the v-Myc avian myelocytomatosis viral oncogene homolog (c-Myc)^17–19^.

## Materials and Methods

### Tissues, animals, cell lines and reagents

Colon biopsies of 6 CD patients and 6 UC patients, as well as 6 subjects without IBD, were obtained from Jinling Hospital, Medical School of Nanjing University and the use of biopsy tissues was approved by the Ethics Research Committee of this institution (gov ID: NCT03578692). An informed consent for the use of tissue was obtained from each tissue donor. All methods were performed in accordance with this institution’s guidelines and regulations. HT29, CT26 and HCoEpiC cells were purchased from Chinese Academy of Sciences (Shanghai, China) and were cultured at 37 °C in a 5% CO_2_ humidified incubator in Dulbecco’s modified Eagle medium (DMEM) (Gibco, CA, USA) or RPMI 1640 medium that contained 10% fetal bovine serum, 100 units/ml of penicillin and 100 μg/ml of streptomycin (Gibco, Gaithersburg, MD, USA). Male C57BL/6 J wild-type mice and CD47-deficiency (CD47^−/−^) mice (T004437) with the same C57 background were obtained from the Animal Model Research Center (Nanjing, China). Animal experimental were approved by the Animal Care Committee of Nanjing University (Nanjing, China) and the procedures were operated according the National Institutes of Health Guide for the Care and Use of Laboratory Animals.

### Wound healing assay

Wound healing assay can assess the migratory ability of cell as described previously^20^. CT26 were planted on 6-well plate and allowed to grow to 90–100% confluence. Then the cell monolayers were wounded by a 200 μl pipette tip and were washed 4 times with PBS. After the cell monolayers were incubated in serum-free DMEM with 100 ng/mL TNFα for 6 h. The distances of cell migrating were quantified.

### EdU staining

Cell proliferation was detected by Cell-Light^TM^ EdU Apollo 488 *In Vitro* Imaging Kit (RiboBio, C103103), according to the instructions. Briefly, cells were treated with 25 mM EdU for 2 h and then fixed by 4% paraformaldehyde. The cells were exposed to 1×Apollo reaction mixture for 30 minutes. Finally, the cells were incubated with DAPI for 15 min and then imaged under Two-photon laser confocal microscope (Lei TCS SP8-MaiTai MP). The signal of Edu^+^cells was analyzed by image J software.

### Crypt isolation and intestinal organoid culture

Intestinal crypt was harvested from intestine of C57BL/6 and CD47^−/−^ mice according to previous reports^21–23^. In brief, intestine was washed in cold Ca^2+^/Mg^2+^-free PBS and cut into small pieces. Then these pieces of intestine were incubated in Cell Dissociation Buffer (absin, 07174- STEMCELL) at 15–25 °C for 15 min. After the cell dissociation was terminated by cold Ca^2+^/Mg^2+^-free PBS containing 0.1% BSA, crypt fractions were collected by passing through 70μm cell strainer repeatedly and purified by centrifugation. Collections were resuspended in ice Advanced DMEM/F-12 (Gibco, 12634010) after washed by PBS and counted under microscope. Crypts collected were re-suspended in 150 μl IntestiCult™ Organoid Growth Medium (STEMCELL, 06005) and 150 μl Matrigel (Corning, 356231) and then seeded in a preheated 24-well plate. Finally, 750 μl IntestiCult™ Organoid Growth Medium was added into each well when Matrigel solidified after incubating at 37 °C for 10 min.

### Epithelial cells isolation

Colon was washed in cold Ca^2+^/Mg^2+^-free PBS and then incubated in 2 mM cold EDTA chelation buffer (100 mM NaCl, 5 mM Na_2_HPO_4_, 2 mM KCl, 40 mM sucrose, 55 mM D-sorbitol, 0.5 mM dithiothreitol, 10 mM KH_2_PO_4_) for 60 min on ice. This tissue fragments were vigorously re-suspended in cold Ca^2+^/Mg^2+^-free PBS by pipette after removal of the chelation buffer. Epithelial cells were collected after passed through a 70-μm cell strainer and centrifugation.

### RNA isolation and quantitative RT-PCR

Total RNA was extracted by TRIzol (Invitrogen, 15596018) according to its instructions. Then the total RNA was reverse-transcribed by AMV reverse transcriptase (Takara, 292–68901). The RNA sample in each reaction was equal and the mRNA expression was normalized by GAPDH. Quantitative RT-PCR was performed using SYBR GREEN I NUCLEIC (Invitrogen, CS7563). Sequences of the primers used were as follows: human CD47, 5′-AGAAGGTGAAACGATCATCGAGC-3′ and 5′-CTCATCCATACCACCGG ATCT-3′; human GAPDH: 5′-CTGGGCTACACTGAGCACC-3′ and 5′-AAGTGGTCGTTGAGGGCAA TG-3′.

### Mouse colitis model

WT C57BL/6 mice (Male, 6–8weeks) were fed with 2% Dextran sulfate sodium (DSS, Sigma-Aldrich, 42867) for 12 days. In another experiment, CD47^−/−^ and WT C57BL/6 mice (Male, 6–8weeks) were fed with 2.5%DSS for 7 days. Mice of both these two experiments were evaluated daily for body weight change and overall body condition including distress and colitic symptoms (wet tool, diarrhea, and bloody diarrhea) and colonic tissue was collected on the indicated day.

### Western Blot

Intestinal tissue and cells were lysed in RIPA lysis buffer (Beyotime, P0013B) with protease inhibitor cocktail (Sigma-Aldrich, P8340), phosphorylase inhibitor (Millipore, 361515) and PMSF (R&D Systems, 4486/50). Then the 4× loading buffer (which is free of SDS for CD47) was added into the cell lysate. The mixture was heated on 60–65 °C for 5 minutes for CD47 while others were heated on 95–99 °C for 8 minutes. Protein levels were analyzed via Western blot using selective antibodies and normalized against the level of GAPDH. The following antibodies were used: anti-c-Myc (Cell Signaling, 2840 s), anti-KLF4 (Cell Signaling, 4083 s), anti-Oct-4A (Cell Signaling, 2840 s), anti-Sox2 (Cell Signaling, 23064 s), anti-CD47 (R&D, BAF1866), anti-GAPDH (Santa Cruz Biotechnology, sc-32233). Quantification of bands was analyzed by software Image J software.

### Transfection of cells with plasmid and siRNA

Cells were transfected at 60% confluence in basal medium using lipofectamine 2000 CD (Invitrogen, 12566014) in Opti-MEM medium (Thermo Fisher Scientific, 31985062) according to its instruction. The final siRNA or plasmid concentration was 1 ng/ml. Conformation of target suppression was provided by Western blot. Mouse siRNA-CD47: 5′-GGAAUGACCUCUUUCACCATT-3′ and 5′-UGGUGAAAGAGG UCAUUCCTT-3′; Mouse siRNA control: 5′-UUCUCCGAACGUGUCACGUTT-3′ and 5′-ACGUGACA CGUUCGGAGAATT-3′. Human Sox2 siRNA target gene sequence: ACCAGCGCATGGACAGTTA; Human c-Myc siRNA target gene sequence: CGACGACCTTCATCAAA; Human Klf4 siRNA target gene sequence: GACCTGGACTTTATTCTCT; Human OCT4 siRNA target gene sequence: CCAGAACTTAG CAGCTTAT. Cells were also transfected with pReceiver-M56-CD47 or control vector PReceiver-M56 plasmids (Fulen Gen Co., Ltd., Guangzhou China).

### Histopathological analysis

Intestinal tissue harvested was fixed in 4% paraformaldehyde for about 24 h at room temperature, dehydrated in different concentrations of ethanol, cleared by xylene and embedded by paraffin. The prepared 3μm colon section slides were de-waxed. Finally, colon sections were stained with hematoxylin and eosin.

### Immunofluorescence

Colon tissues were frozen in OCT, and then sliced by Leica CM1950 at 6μm thickness, fixed in 4% paraformaldehyde, blocked with 10% FBS-PBS for 30 min, and incubated with anti-CD47 (R&D, BAF186) and anti-E-cadherin (Abcam, 76055) primary antibodies overnight at 4 °C. After washing, the slides were incubated with fluorophore-conjugated secondary antibodies for 1 h, and the antibodies included Alexa Fluor^TM^488 goat anti-mouse (Invitrogen, A11001), Alexa Fluor^TM^594 donkey anti-rabbit (Invitrogen, A21207), Alexa Fluor^TM^488 donkey anti-rabbit (Invitrogen, A21206) and Alexa Fluor^TM^594 goat anti-mouse (Invitrogen, A11005) antibodies^24^. Finally, the slides were incubated with DAPI for 15 min and then imaged under Two-photon laser confocal microscope. The fluorescence intensity of images was analyzed by image J software.

### Flow cytometric assays

HT29 or HCoEpiC cells were plated onto 6-well plates for overnight culture and then treated with IFNγ, TNFα, IL-17 (100 ng/ml) for 6 h, respectively. The harvested cells were washed twice with ice-cold PBS and then were incubated with blocking solution for 15 minutes. After that, FITC-conjugated CD47 antibody (Santa Cruz Biotechnology, sc-21786) was added and incubated for 30 minutes at 4 °C. Following several washes with ice-cold PBS, the cells were analyzed using flow cytometer (BD, Franklin Lake, NJ, USA) with Win MDI 2.9 software.

### Statistical analysis

The data were analyzed using the GraphPad Prism 6. Data are presented as the means ± standard error of the mean (SEM). When only two groups were compared, Student’s *t*-test was used. More than two group differences were analyzed by ANOVA with Tukey-Kramer test. A *P*–value < 0.05 was considered statistically significant.

## Results

### Upregulation of CD47 in human intestinal epithelium in UC and CD patients and in mouse intestinal epithelium during DSS-induced experimental colitis

**Figure 1:**
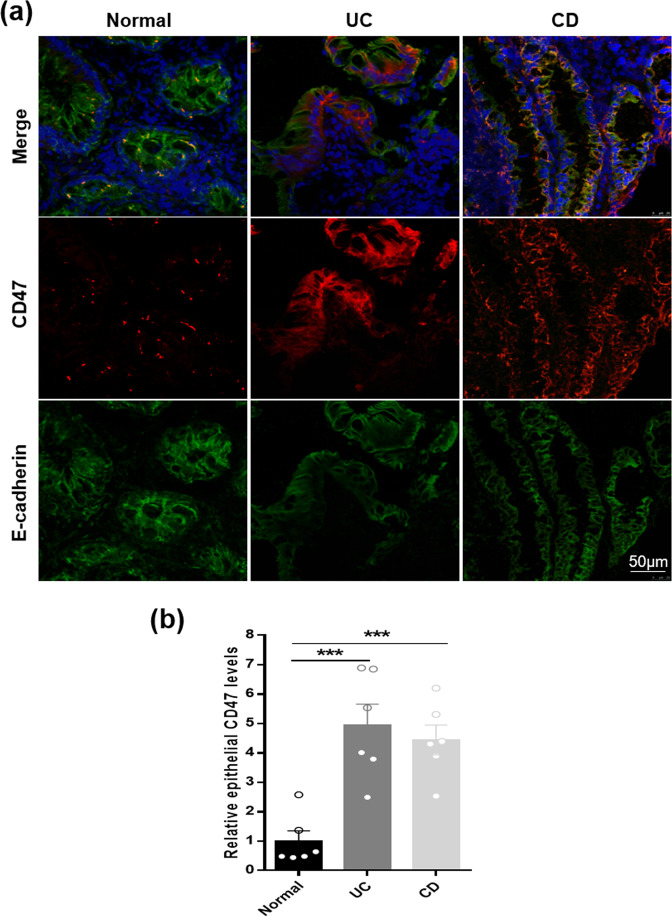
CD47 upregulation in human intestinal epithelium from IBD patients. (**a**) CD47 immunostaining (red) and E-cadherin (green) doubling staining of colonic tissue section. Nuclei were counterstained with DAPI (blue) (n = 6, per). (**b**) Statistical analysis of CD47 expression in intestinal epithelium from non-IBD control (n = 6), patients with UC (n = 6) and patients with CD (n = 6). ***P < 0.001 versus non-IBD control.

To examine CD47 level in human intestinal epithelium under IBD condition, we collected colonoscopic biopsies (6 each) from Crohn’s diseases (CD), ulcerative colitis (UC) patients and subjects without chronic intestinal inflammatory condition. In this experiment, we co-stained tissue sections with antibodies against CD47 and epithelial marker E-cadherin. As shown in Fig. 1, compared to control subjects without intestinal inflammatory condition, patients with active CD and UC both had a significantly upregulated CD47 expression in intestinal epithelium. In line with previous findings of large amount of infiltration of inflammatory immune cells in the intestine tissue of CD and UC patients^25^, we also observed more infiltration of CD11b^+^ immune cells (arrowheads) in CD and UC patients’ tissue sections compared to control tissue sections (Supplementary Fig. S1). Although the infiltration of CD47 positive immune cells might increase the total CD47 level in colonic tissues of CD and UC patients, it did not contribute to the enhancement of CD47 expression in the intestinal epithelium (marked with white frame).

**Figure 2:**
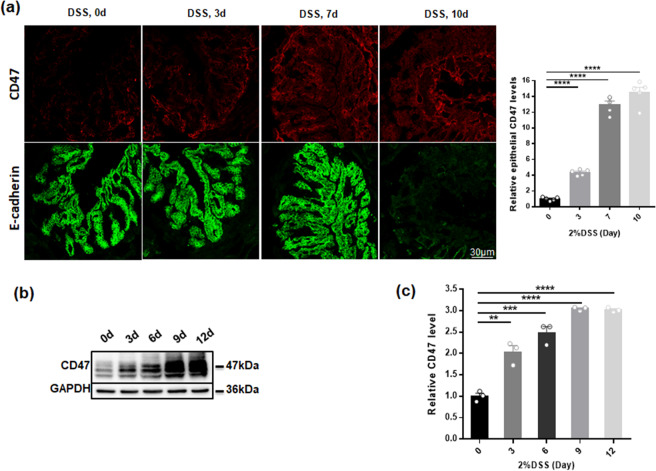
CD47 upregulation in mouse intestinal epithelium during DSS-induced experimental colitis. The mice (n = 20) were treated with 2%DSS for various time points. (**a**) CD47 immunostaining (red) and E-cadherin (green) doubling staining of colonic section in day 0, day 3, day 7, day 10 (n = 5). (**b,c**) Western blotting analysis of CD47 expression in dissected intestinal epithelium from DSS-treated mice on day 0, day 3, day 6, day 9 and day 12 post-treatment (n = 3). **P < 0.01, ***P < 0.001, ****P < 0.0001 versus day 0.

Similar to observations in intestinal tissue samples from IBD patients, CD47 upregulation in IECs was also detected in DSS-induced murine experimental colitis model. The disease symptoms included rapid loss of body weight (Supplementary Fig. S2a), the significant increase of disease activity index (DAI) score (Supplementary Fig. S2b) and inflammatory cell infiltration and apparent damage in H&E staining (Supplementary Fig. S2c). Mice displayed body weight loss and colitis symptoms (diarrhea) from day 3 post-DSS treatment. The colitic condition was exacerbated around day 7, and the mice were eventually sacrificed on days 9 or 12. As shown in Fig. 2, the kinetic of CD47 induction in mouse IECs was determined by immunofluorescence labeling and western blotting on different days post-DSS treatment. Both immunofluorescence labeling (Fig. 2a) and western blotting (Fig. 2b,c) showed that CD47 expression in mouse intestinal epithelia was markedly increased after 7 days DSS treatment. These results suggest that upregulation of intestinal epithelial CD47 may contribute to the colitis development.

### Inflammatory cytokines enhance intestinal epithelial cell CD47 expression

**Figure 3:**
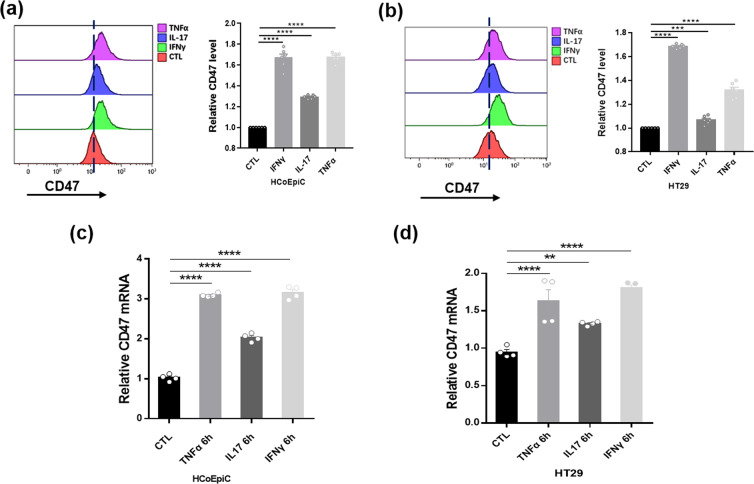
CD47 upregulation in human IECs by inflammatory cytokine treatment. (**a,b**) Analysis of CD47 expression in HCoEpiC (**a**) and HT29 (**b**) cells with or without inflammatory cytokine treatment (100 ng/ml, 6 h). CD47 was labeled with anti-CD47 Abs followed by FITC. (c, d) Relative mRNA levels of CD47in HCoEpiC (**c**) and HT29 (**d**) cells with or without inflammatory cytokine treatment. *P < 0.05, **P < 0.01, ***P < 0.001, ****P < 0.0001 versus control.

To confirm the upregulation of CD47 expression in colonic epithelial cells under inflammatory condition, we treated human intestinal epithelial cells, HT29 and HCoEpiC cells, with inflammatory cytokines IFNγ, TNFα or IL-17 (each 100 ng/ml) for 6 h. Flow cytometry results showed that CD47 expression in human IECs was significantly increased after inflammatory cytokine treatment (Fig. 3a,b). The elevation of CD47 mRNA level by inflammatory cytokines was also detected by qRT-PCR assay (Fig. 3c,d).

### CD47 deficient mice resist to DSS colitis

**Figure 4:**
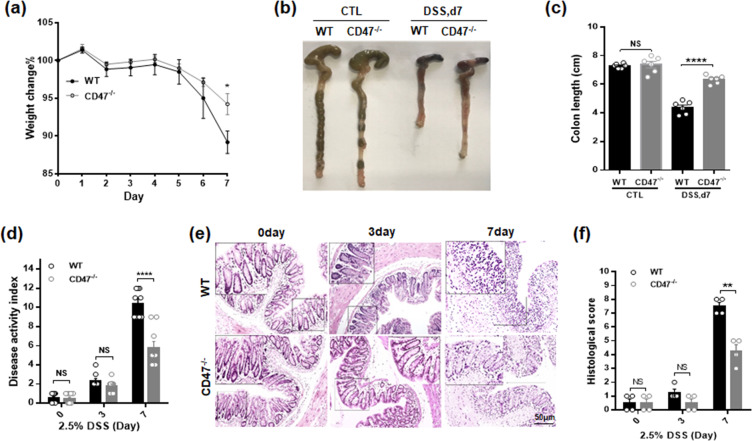
CD47 deficiency protects the mice from DSS-induced experimental colitis. Mice (male, 20–22 g, n = 6) were given 2.5% DSS in drinking water for 7 day. (**a**) Bodyweight loss. (**b,c**) Colin length. (**d**) Disease activity index. (**e,f**) Histology of colon tissue (H&E staining) (**e**) and histological scoring (**f**). NS, no significance, ****P < 0.0001versus WT mice.

To further examine the inflammatory condition in WT and CD47^−/−^ mice during intestinal inflammatory injures, mice were treated with 2.5% DSS for 7 days. Deficiency of CD47 in KO mice was confirmed by western blotting (Supplementary Fig. S3). In agreement with previous reports^15^, we found that CD47^−/−^ mice are more resistant against DSS-experimental colitis. Compared to WT littermates, CD47^−/−^ mice exhibited less bodyweight drop (Fig. 4a) and shortening of the intestine (Fig. 4b,c) induced by DSS treatment. Disease activity index (Fig. 4d), tissue section examination (Fig. 4e) and histological scoring (Fig. 4f) also clearly showed that CD47^−/−^ mice displayed less intestinal epithelial tissue damage during DSS-induced experimental colitis.

### CD47 deficiency promotes the proliferation and wound healing of IECs

**Figure 5:**
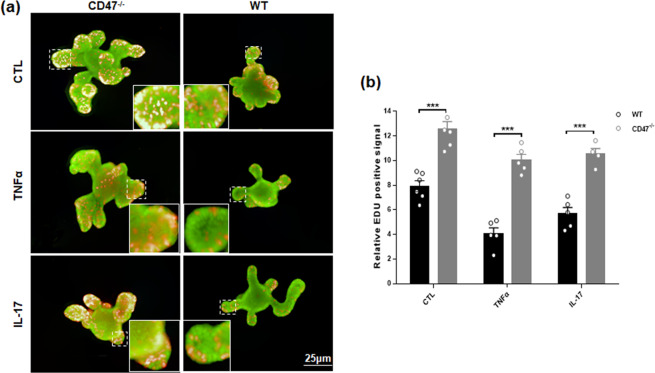
Negativecorrelation between the intestinal epithelial cell CD47 expression level and the cell proliferation capacity. (**a**) Organoids were stained with EDU (red). Nuclei were counterstained with DAPI (green). (**b**) EDU assay of proliferation of intestinal epithelial cells isolated from WT and CD47^−/−^ mice. Cells were treated with or without various inflammatory cytokines (n = 5). ***P < 0.001.

To explore the mechanism underlying the protection of CD47 deficiency in intestinal epithelium against colitic injury, we isolated intestinal organoid from WT and CD47^−/−^ mouse colon and cultured in Matrigel as previous described^22^. During culture, intestinal organoid was treated with or without IL-17 or TNFα (each 100 ng/ml, 24 h)^26,27^. As shown in Fig. 5a, CD47^−/−^ intestinal organoids displayed significantly more proliferation in the crypt than WT intestinal organoids in the presence or absence of inflammatory cytokines. Quantitative analysis indicated that the numbers of Edu-positive cells in the crypt were significantly higher in organoids isolated from CD47^−/−^ mice than those from WT mice with or without inflammatory cytokine treatment (Fig. 5b). This result indicates that CD47 deficiency has a positive role in intestinal epithelial cell proliferation.

**Figure 6:**
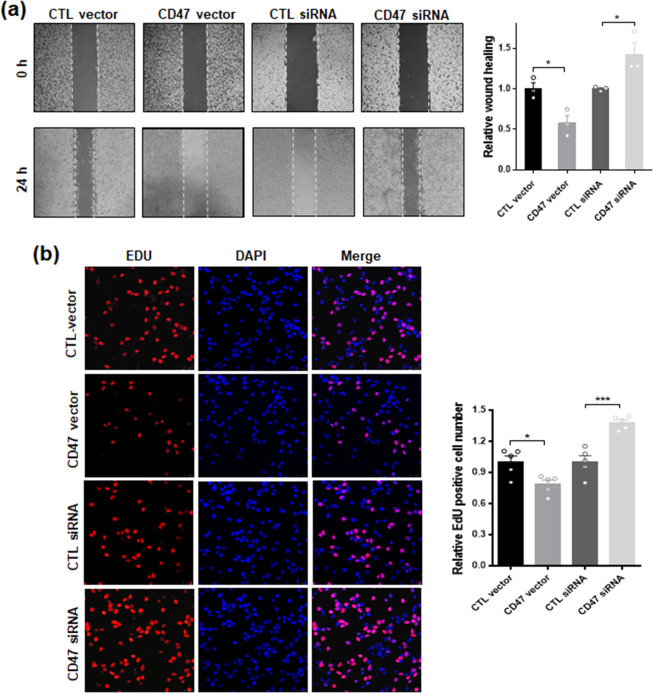
Intestinal epithelil cell CD47 inhibits cell wound healing and proliferation processes. (**a**) Overexpression or depletion of CT26 epithelial cells CD47 inhibits or promotes cell wound healing, respectively. (**b**) EDU assay of proliferation of CT26 cells. Cells were overexpressed or depleted CD47 via transfecting with CD47-expressing vector or CD47 siRNA. *P < 0.05, ***P < 0.001.

Next we performed cell wound healing assay to test the effect of CD47 on intestinal epithelial cell migration capacity^28^. In this experiment, CT26 cells were cultured on tissue culture plated to confluent. Prior to scratch using comb, cells were transfected with CD47-expression vector or CD47 siRNA oligonucleotides, as well as control vector or control oligonucleotide, to increase or decrease the cellular level of CD47 expression (Supplementary Fig. S4a). As shown in Fig. 6a, the wound closure of CT26 cells at 24 h post-injury was strongly inhibited by transfection with CD47 vector compared to that in control vector-infected cells, while depletion of cellular CD47 via CD47 siRNA significantly enhanced the wound healing of CT26 cells compared to that in cells treated with control siRNA. EDU assay also demonstrated that overexpression of CD47 or depletion of CD47 suppressed or promoted CT26 cells proliferation, respectively (Fig. 6b). Similar results of CD47-deficiency promoting cell proliferation and wound healing were observed in intestinal epithelial HT29 cells (Supplementary Fig. S5).

### CD47 **reduces the expression of Oct4, Klf4, Sox2 and c-Myc in intestinal epithelial cells**

**Figure 7:**
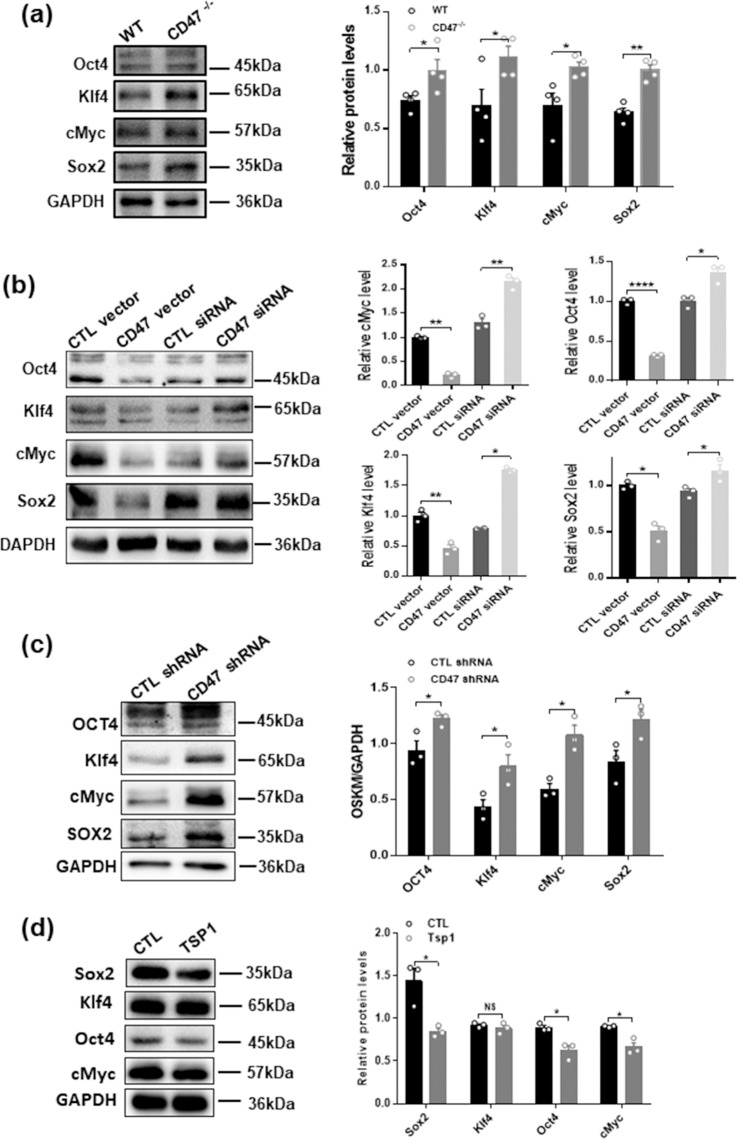
CD47 deficiency upregulates the expression of OSKM in intestinal epithelial cells. (**a**) Western blot analysis of OSKM expression in intestinal epithelial cells from WT and CD47^−/−^ mice treated with 2.5% DSS. (**b**) Overexpression or depletion of CD47 in CT26 cells reduced or enhanced the expression of OSKM factors, respectively. (**c**) Depletion of CD47 in HT29 cells enhanced the expression of OSKM. (**d**) Exogenous TSP1 can downregulate expression of Sox2, Oct4 and cMyc in HT29 cell line, but not Klf4. NS, no significance, *P < 0.05, **P < 0.01, ****P < 0.0001 versus WT or control.

Recent studies by Rogers *et al*. implicated that CD47 might serve as an negative regulator of several essential cell pro-survival transcriptional factors, including Oct4, Klf4, Sox2 and c-Myc (termed as OKSM in abbreviation)^14^. To test whether inhibition of OKSM expression by CD47 can serve as a mechanism through which CD47 suppresses intestinal epithelial proliferation and wound healing processes, we examined the levels of these 4 transcriptional factors in intestinal epithelium isolated from WT and CD47^−/−^ mice using Western blot analysis. As shown in Fig. 7a, levels of Oct4, Klf4, Sox2 and c-Myc in isolated intestinal epithelium were significantly higher in CD47^−/−^ mice than those in WT mice. Upregulation of intestinal epithelial Oct4, Klf4, Sox2 and c-Myc levels in CD47 deficient mice was confirmed by directly increasing or depleting CD47 in CT26 cells. In this experiment, CT26 cells were overexpressed with CD47 vector or CD47 siRNA to increase or deplete cellular CD47. As can be seen, levels of cellular Oct4, Klf4, Sox2 and c-Myc were significantly reduced in cells overexpressed with CD47 but enhanced in cells with CD47 depletion (Fig. 7b). The increase of OSKM levels caused by CD47 knockdown was further validated in intestinal epithelial HT29 cells (Fig. 7c) and knockdown of each OSKM factor can reverse the enhancement of cell proliferation induced by CD47 deficiency (Supplementary Fig. S5). As thrombospondin-1 (TSP1) can also suppresse OSKM via binding CD47 in renal tubular epithelial cells^12–14^, we incubated HT29 cells with exogenous TSP1 and found that TSP1 downregulated the expression of Sox2, Oct4 and cMyc but not Klf4 in HT29 cells (Fig. 7d).

## Discussion

In this study, we have demonstrated that epithelial cell CD47 contributes to the developing of IBD in human and mice. CD47 expression in the colon is strongly induced in patients with UC and CD and in mouse with DSS-induced experimental colitis. Results from cell cultures also showed the increase of CD47 expression after treatment with proinflammatory cytokines. CD47 deficiency specific in mouse intestinal epithelia premotes the proliferation and self-renew of IEC following the DSS-induced damage maintains the intergrity of the epithelial barrier.

Besides serving as SIRPα liangd to initiate inhibitory signal for macrophage phagocytosis against self cells^15,29–31^, CD47 has been shown as a suppressor for tumor metastasis, and targeting it presents an potential and effective therapeutic strategy^32–34^. As CD47 is tightly associated with a number of aggressive cancers, CD47 may serve as a positive predictive marker of cancer progression and metastasis^35–37^. Our study demonstrated that intestinal epithelial cell CD47 was also markedly upregulated under inflammatory condition, and its expression was positively correlated with the severity of inflammatory colitis. From this angle of view, CD47 may serve as a new diagnostic marker and therapeutic target for IBD particularly at early stage. Our results further indicated that CD47 deficiency protects mice from DSS-induced acute colitis and accelerates IEC recovery from damage induced by DSS treatment, suggesting that CD47 serves as a negative regulator for intestinal epithelial cell wound healing. Given that CD47 deficiency significantly relieved DSS-induced colitis, downregulation of CD47 expression in the IEC may improve the condition of IBD. The notion that CD47 downregulation can promote cell survival and renewal process is supported by previous reports from different groups. Lin *et al*.^38^ showed that CD47 blockade reduces ischemia-reperfusion injury and improves outcomes in a rat kidney transplant model. Employing skin graft model, Isenberg *et al*.^39^ found that suppressing CD47 expression drastically increases graft survival. The study by Soto-Pantoja *et al*.^40^ demonstrated that CD47 deficiency could protect cells and tissues against radiation damage by activating autophagy pathway. Moreover, in agreement with previous report by Bian *et al*.^15^ that CD47 deficiency protected mouse colitis induced by low dosage of DSS, we also found that CD47 deficiency failed to rescue mouse colonic damage induced by 4% DSS treatment. These results suggest that protection by CD47 deficiency may be limited against severely colonic damage by high dosage of DSS.

Mechanistic studies further demonstrated that CD47 signaling pathways may control cellular differentiation and responses to stress such as ionizing radiation through g^11,41^. Recent studies by Rogers *et al*. implicated that CD47 might be a negative regulator in the expression of cell pro-survival transcriptional factors, including Oct4, Klf4, Sox2 and c-Myc^14^. Testing the primary intestinal epithelial cells isolated from WT and CD47^−/−^ mice, we found that levels of Oct4, Klf4, Sox2 and c-Myc in isolated intestinal epithelium from CD47^−/−^ mice were indeed markedly higher than those in WT mice. In support of this, overexpression or depletion of CD47 in intestinal epithelial cell lines suppressed or enhanced the expression of cellular Oct4, Klf4, Sox2 and c-Myc, respectively. These results may suggest that CD47 modulates intestinal epithelial cells self-renewal through negatively regulating the expression of cell pro-survival transcriptional factors OKSM, which have been widely shown to activate cell dedifferentiation^18,42^ and proliferation^43,44^.

In order to explore the mechanism underlying the protection of CD47 deficiency onintestinal epithelial cells against DSS-induced injures, we established a stable system of intestinal organoids culture. The results of the staining with EDU of the intestinal organoids before and after the treatment of TNFα or IL-17 both indicated that CD47 deficiency can improve the proliferation and self-renewal of the IECs. Based on these results, we conclude that CD47 deficiency relieves the colitis mainly by enhancing the proliferation of the IEC which are the keys to cell recovery following the damage and cell apoptosis induced by DSS or proinflammatory cytokines. However, we should mention a very recent study by Reid *et al*., finding that IEC-expressing CD47 is in fact required for mucosal repair^45^. Employing similar IEC-psecific CD47-KO mouse model, they concluded that the defective wound repair in CD47 deficient cells is linked to decreased epithelial β1 integrin and focal adhesion signaling, two essential factors for cell migration. Different from our study that focused on IEC proliferation within 7 days of DSS-induced damage, the study by Reid *et al*. monitored IEC wound healing process (in which cell migration plays an important part) in 22–26 days along DSS-water-DSS cycle. The contraversial results from these two studies may suggest that the roles of CD47 in regulating intestinal epeithelial cell proliferation and migration process are different.

In conclusion, our resultshave demonstrated that CD47 deficiency protects intestinal epithelium against colitis by promoting proliferation and self-renewal of IEC and maintaining the intergrity of the epithelial barrier.

## Supplementary information

Supplementary information.
