# Supplementary material for: CD47 is a negative regulator of intestinal epithelial cell self-renewal following DSS-induced experimental colitis
Source: Sci Rep. 2020 Jun 23;10:10180. doi: 10.1038/s41598-020-67152-w (PMC7311394; doi:10.1038/s41598-020-67152-w)
Supplement: Supplementary file 1 — Supplementary information. [file 41598_2020_67152_MOESM1_ESM.pdf]

CD47 is a negative regulator of intestinal epithelial cell self-renewal following DSS-induced experimental colitis

Yueqing He[1*], Xinlei Sun[1*], Weiwei Rong[1*], Rong Yang[1], Hongwei Liang[1], Ying Qi[3], Limin Li[1,2¶] and Ke Zen[1,2¶]

File list

1. Supplementary Figure S1
2. Supplementary Figure S2
3. Supplementary Figure S3
4. Supplementary Figure S4
5. Supplementary Figure S5
6. Supplementary Figure S6 (raw gel/blots)

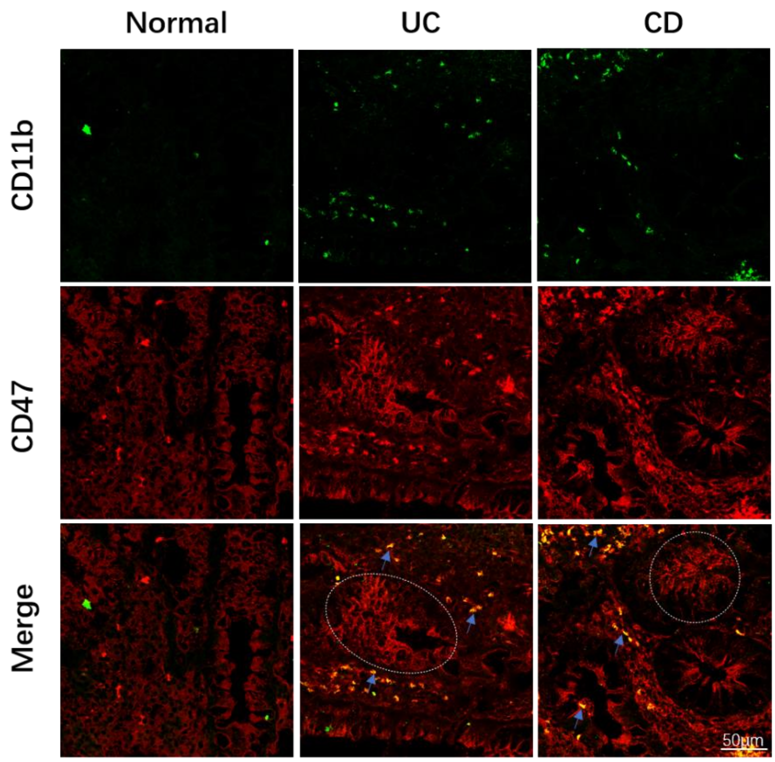

Supplementary Figure S1. Increased CD11b^{+} immune cell infiltration in UC and CD patients' intestine (n=5). Note that infiltration of CD47 positive immune cells (arrowheads) may increase the total CD47 level of tissue, but does not contribute to the increase of CD47 expression in colonic epithelium (white frame).

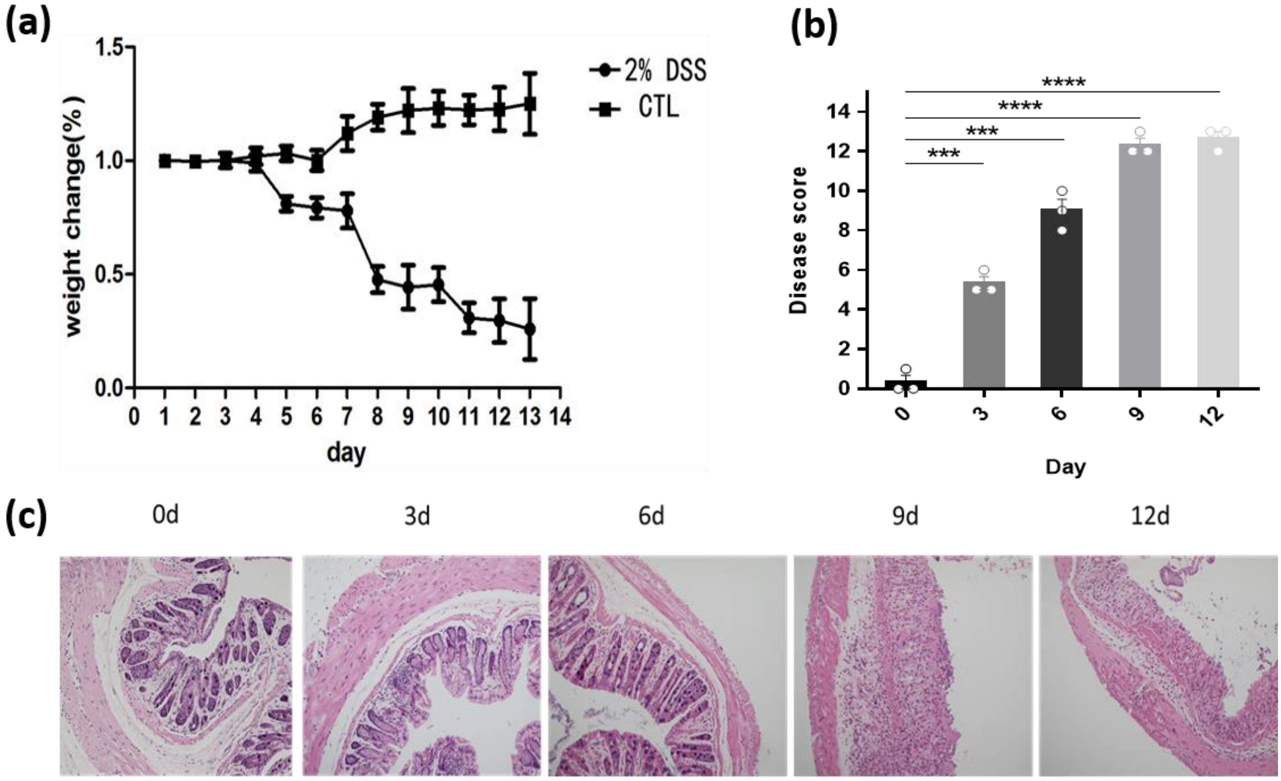

Supplementary Figure S2. The mice were treated with 2% DSS for 12 days followed by regular drinking water. (a) Body weight loss and (b) disease activity index (DAI) scores. (c) Histology of colon tissue (H&E staining). ***P<0.001, ****P<0.0001 versus 0 day.

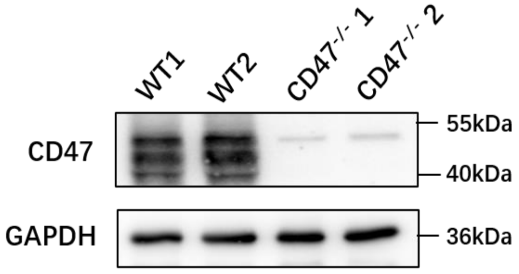

Supplementary Figure S3. Confirmation of CD47 knockout by Western blotting.

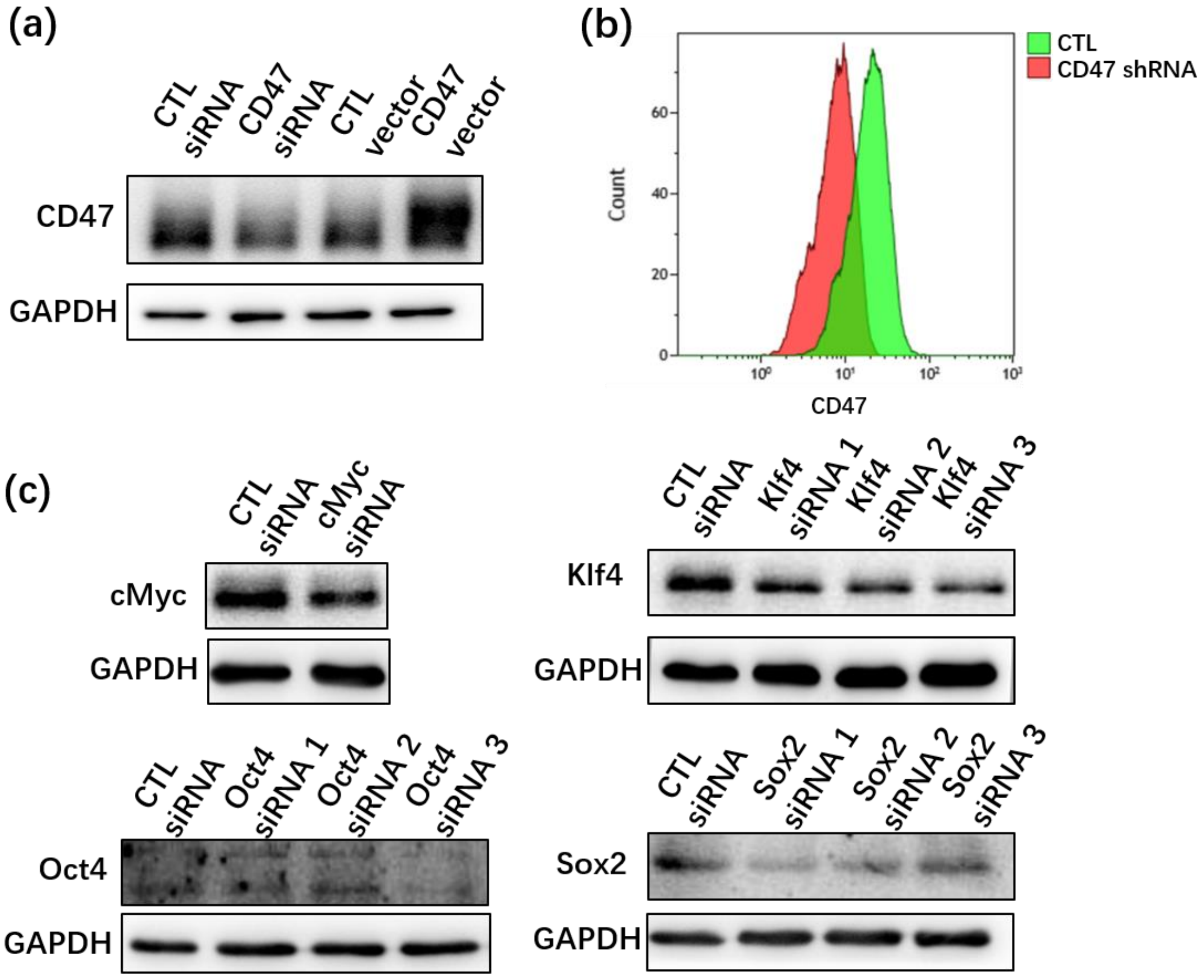

Supplementary Figure S4. Detection the efficiency of siRNA and shRNA oligonucleotides and expression plasmid. (a) Increase or decrease the level of CD47 expression in CT26 cells tested by Western Blot. (b) Knockdown of CD47 in HT29 cells by CD47 shRNA tested by Flow Cytometry. (c) Western blot analysis showed that cMyc siRNA, Oct4 siRNA3, Klf4 siRNA3 and Sox2 siRNA2 were efficient in inhibiting the expression of the respective genes in HT29 cells.

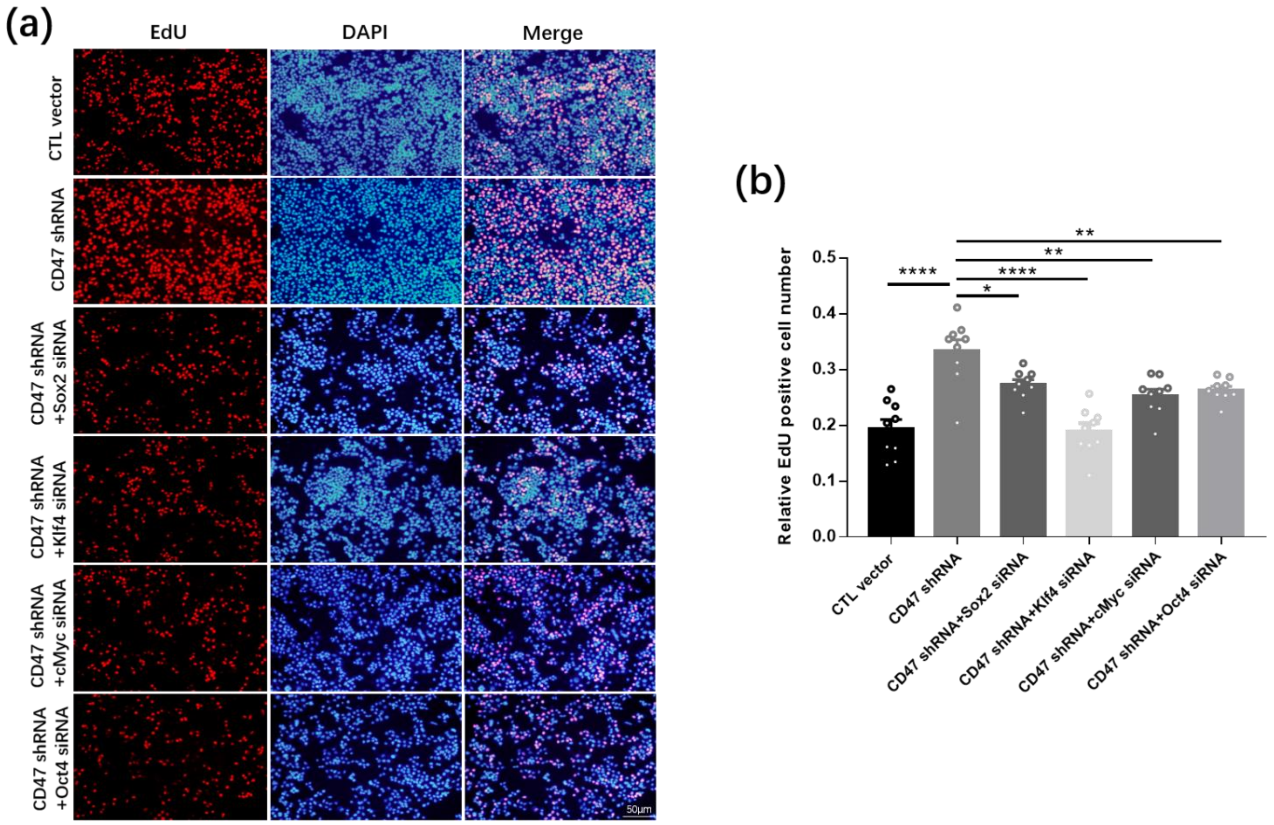

Supplementary Figure S5. Knocking down OSKM separately reverses the enhancement of cell proliferation capacity induced by CD47 knockdown. After CD47 knockdown in HT29 cells, each OSKM factor was silenced by transfection of specific siRNA, respectively. Cell proliferation was assessed by EdU assay. (a) Representative images of cell proliferation. (b) The analysis of EdU-positive cell number. *P<0.05, **P<0.01, ****P<0.0001 versus control or CD47 shRNA.

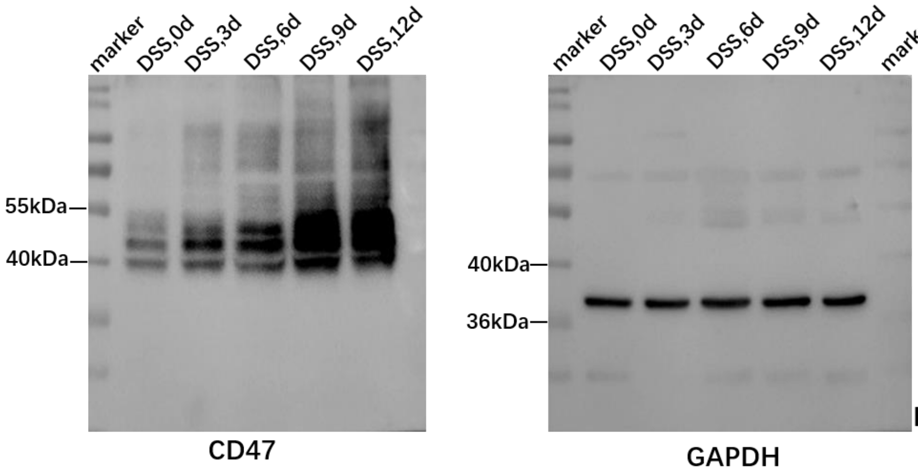
marker
DSS,0d
DSS,3d
DSS,6d
DSS,9d
DSS,12d
55kDa
40kDa
CD47
marker
DSS,0d
DSS,3d
DSS,6d
DSS,9d
DSS,12d
marker
40kDa
36kDa
GAPDH

Fig.2(b)

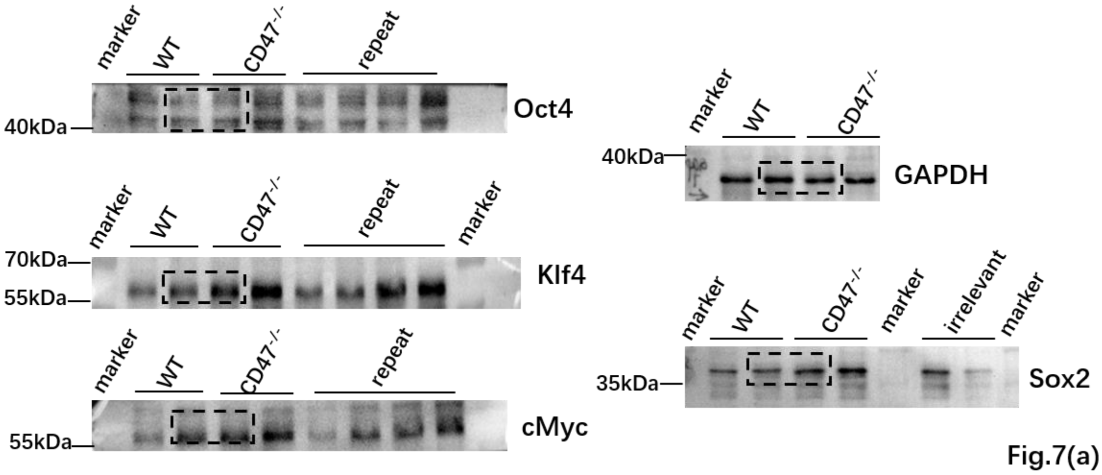
marker
WT
CD47-/-
repeat
40kDa
Oct4
marker
WT
CD47-/-
repeat
marker
70kDa
55kDa
Klf4
marker
WT
CD47-/-
repeat
55kDa
cMyc
marker
WT
CD47-/-
40kDa
GAPDH
marker
WT
CD47-/-
marker
irrelevant
marker
35kDa
Sox2

Fig.7(a)

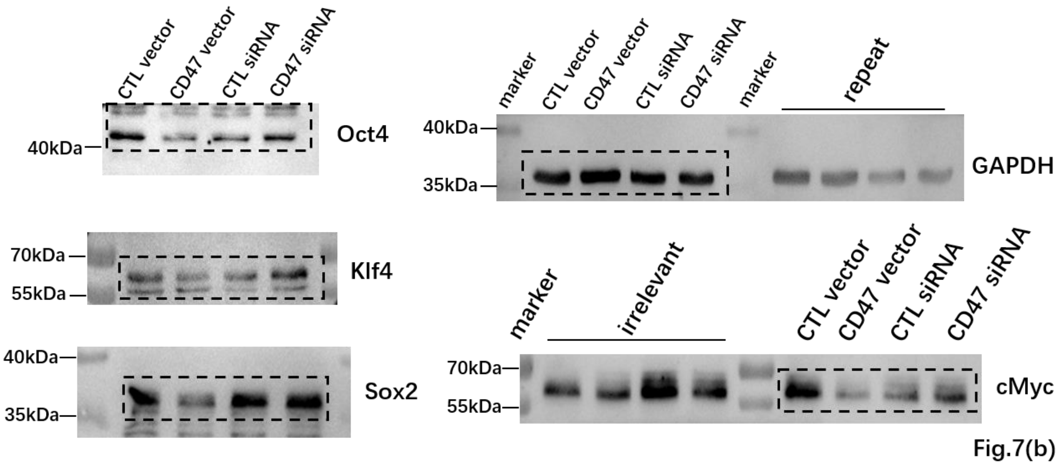

Fig.7(b)

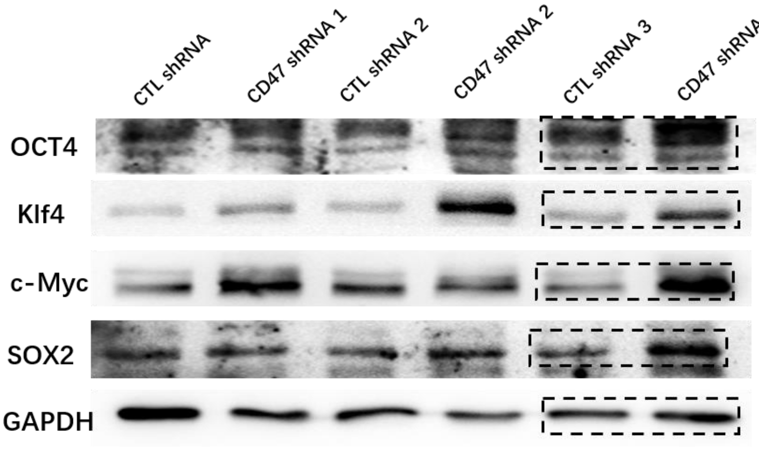

Fig.7(c)

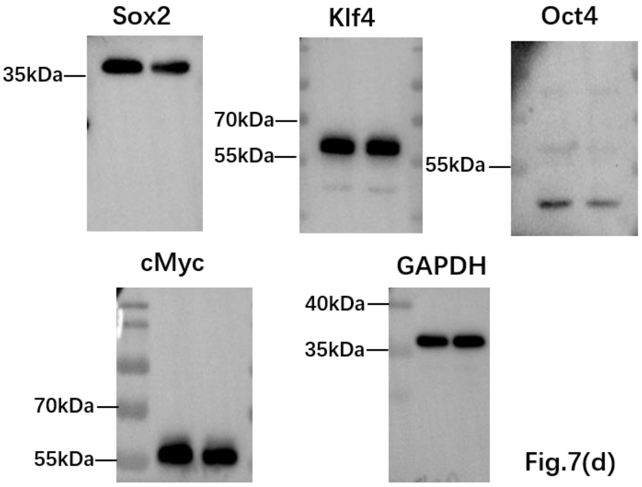

Fig.7(d)

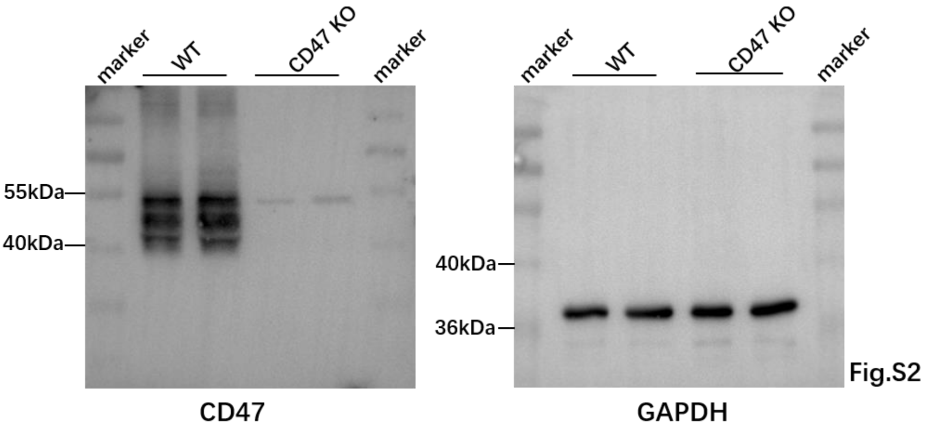

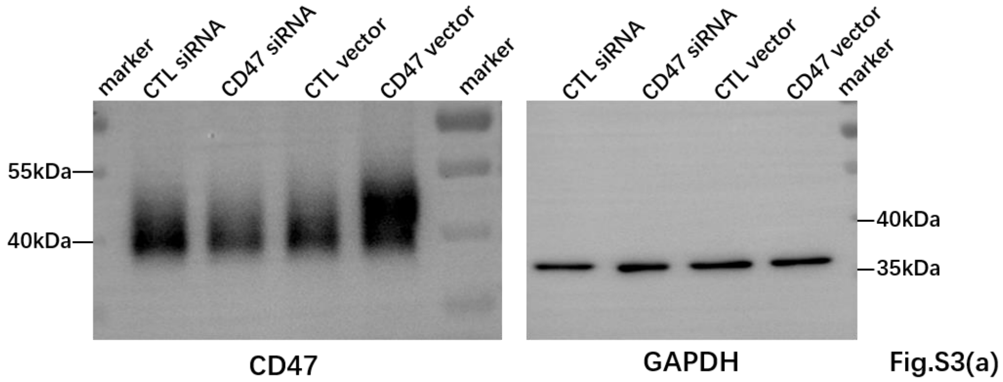

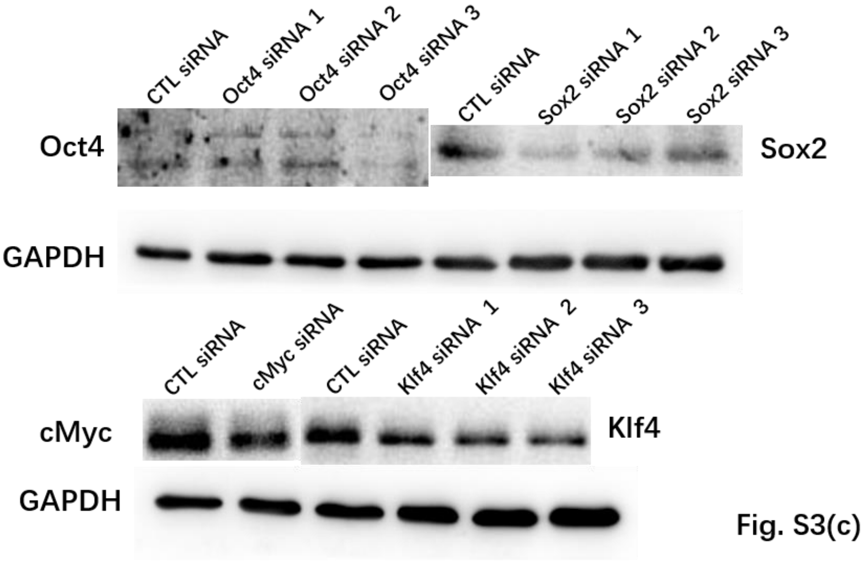

Supplementary Figure S6. Raw gels and blots for all the figures.
